# Design and psychometric properties of a tool to assess the knowledge, attitude and practice of health care workers for infodemic management (KAPIM-Tool)

**DOI:** 10.1186/s12913-023-09822-9

**Published:** 2023-08-08

**Authors:** Arezoo Dehghani, Fatemeh Zarei

**Affiliations:** 1 Department of Health in Emergencies and Disasters, School of Public Health and Safety, Shahid-Beheshti University of Medical Sciences, Tehran, Iran; 2 Department of Health Education and Health Promotion, Faculty of Medical Sciences, Tarbiat Modares University, Tehran, Iran

**Keywords:** Infodemic, Health Personnel, Knowledge, Attitude, Behavior

## Abstract

**Background:**

Infodemics, defined as the rapid spread of misinformation during an epidemic or pandemic, can have serious public health consequences. Healthcare workers(HCWs) play a critical role in managing infodemics, but their knowledge, attitudes, and practices(KAP) related to infodemic management are not well understood. This study aimed to design and validate a tool to assess healthcare workers’ KAP related to infodemic management.

**Methods:**

The knowledge, attitude, and practice of HCWs for the infodemic management assessment tool were designed through exploratory factor analysis. At first, primary items were extracted through two separate studies (face-to-face interviews with 17 participants and a systematic review). Then Face validity, Content validity, and Construct validity were done with the 15 participants of healthcare workers who had sufficient knowledge and experience. The content validity ratio (CVR) and content validity index (CVI) was checked for each item. The construct validity of the tool was also calculated through exploratory factor analysis with the participation of 250 healthcare workers (6.25 participants per item). The intraclass correlation coefficient (ICC), and Cronbach’s alpha was calculated to evaluate the reliability of the findings using IBM SPSS Statistics V21.0.

**Results:**

The primary KAPIM (Knowledge, Attitude, and Practice) of healthcare workers in (the Infodemics Management) tool has 53 items, in content, face, and construct validity 13 items were removed. Factor analysis revealed three factors: knowledge (24 items), attitudes (8 items), and practice (8 items). The overall reliability of the tool was reported as adequate with a Cronbach’s alpha of 0.905. The ICC of the entire tool was calculated as 0.827.

**Conclusion:**

The KAPIM tool is a valid and reliable tool for assessing healthcare workers’ knowledge, attitudes, and practices related to infodemic management with 40 items. The tool can inform targeted interventions to improve healthcare workers’ preparedness and response to infodemics.

## Introduction

The COVID-19 pandemic has brought about unprecedented challenges worldwide, not only for healthcare systems but also for individuals trying to keep themselves informed and safe. In this context, the infodemic - the rapid spread of misinformation, disinformation, and rumors about the pandemic through various channels, including social media - has emerged as a significant threat to public health [1]. Health professionals are in a unique position to address and manage this infodemic through their knowledge, attitude, and practice(KAP).

The term infodemiology was first coined in 2002, and concerns about the spread of misinformation are almost as old as the World Wide Web itself [2]. In today’s rapidly changing world, in crises, especially the COVID-19 pandemic, some problems such as the rapid rate of dissemination of new scientific information and the inability of researchers, policy makers, journalists, health service providers and ordinary citizens to keep up with the realities It is rapidly changing. In other words, the current pandemic is partly a challenge to filter (in real time) the large amount of information released on a daily basis and share roumors and infodemics [3]. Infodemiology has gained recognition as a crucial field of study during pandemics by public health organizations and the WHO (World Health Organization) [4]. ensuring accurate and timely knowledge translation while minimizing distorting factors such as political or commercial influences [5].

The importance of infodemic and rumor management in the health care sector has been increasingly recognized in recent years, particularly in the context of the COVID-19 pandemic [6]. Infodemic refers to the rapid and far-reaching spread of information and misinformation during an epidemic, pandemic, or other health emergency [7]. The infodemic can have serious consequences, as people may act on false information or become overwhelmed with contradictory information, leading to confusion and panic. Rumors, which are defined as “unverified information that is circulating without confirmation of truth or accuracy” [7, 8], are a major component of the infodemic.Health professionals must address rumors promptly, accurately, and transparently to prevent misinformation from spreading and mitigate the consequences of the infodemic [9]. This requires a comprehensive understanding of the factors that contribute to rumor transmission, including cultural, social, and psychological factors, as well as knowledge of effective communication strategies.

Several studies have investigated the KAP of healthcare workers regarding rumor management and infodemic, highlighting the need for interventions to improve these skills [10–12]. Moreover, a few tools have been developed to assess healthcare workers’ KAP regarding infodemic management. For instance, a study by Alawa and et al. in 2021 [13] developed and validated a 13-item questionnaire to assess healthcare professionals’ perception of the infodemic. Another study by Limaye RJ and et al. in 2020 [14] developed a tool to assess COVID-19-related knowledge, attitudes, and practices among healthcare workers in India. While these studies are a step in the right direction, they focus on specific aspects of infodemic and rumor management and may not capture the breadth of knowledge and skills required to effectively manage the infodemic.

In addition, some of these tools have not been validated or standardized, limiting their generalizability and utility in different contexts. Thus, there is a need for a reliable and valid tool to assess healthcare workers’ KAP regarding infodemic management. Therefore, the present study aims to address the need for a reliable and comprehensive tool to assess healthcare workers’ KAP regarding infodemic management.

## Methods

### Design and sample

This is a mixed-method study that aimed to design and assess the psychometric properties of a tool, called KAPIM-Tool, to evaluate healthcare workers’ KAP in managing infodemics. Data collection was conducted between January and March 2023. The sample size was determined based on the assumption of having at least 3–10 participants per item [15], with a 10% drop rate for incomplete questionnaires. The inclusion criteria for participation in the study were being Iranian healthcare workers(HCWs) with a minimum of 2 years of work experience in the healthcare system. There were no other exclusion criteria for participation. Participants were recruited through virtual social networks, and they were asked to complete an online questionnaire to help with rumor and infodemic management during pandemics such as COVID-19. The survey was conducted anonymously, and the contact information of the participants was kept confidential. The study was approved by the ethical committee of Iran University of Medical Sciences, Tehran, Iran, with the ethics code IR.IUMS.REC.1399.474.

### Questionnaire desining

The development of the KAPIM-Tool is divided into two main stages: (1) Creating the questionnaire, and (2) Assessing the psychometric properties of the questionnaire.

#### Creating the questionnaire

##### Contextual item generation based on literature review

At this phase, a literature review was conducted to identify the components and concepts related to knowledge, attitude and practice in the field of infodemic management. The search was done in Web of Science, Scopus, PubMed and Google Scholar search engine without any time limitation. All the studies were entered without any restrictions and the components were extracted by reviewing the full text of the articles.

##### Contextual item generation based on qualitative phase

To elucidate the experiences and perceptions of healthcare workers (HCWs) and to collect data, an initial three in-depth unstructured interviews were conducted. After extracting the main concepts, an interview guide was created and subsequent interviews with participants were conducted in a semi-structured manner. A qualitative-directed content analysis, based on the Knowledge, Attitude, and Practice (KAP) model, was designed. Participants were purposefully selected from HCWs and, after obtaining written consent, general questions were asked about their experience with infodemic management, their definition of the concept, and more detailed questions about their perceptions and management of rumors and infodemics. Probing questions were also employed to further explore participants’ experiences.

The data collection tool was the first author (AD), an experienced researcher in the field of risk communication. The interviewer, under equal conditions and after informing the interviewee about the objectives of the study and obtaining their trust and informed consent to participate, began with the general question “How are rumors related to health subjects managed?“ and continued using probing questions. In addition to the interview, pens, paper, and note-taking were used to record participants’ voices (with their permission).

Graneheim and Lundman’s inductive content analysis method was used to analyze the data obtained from the interviews. Data collection and analysis were performed simultaneously; after each interview, the recorded voice was transcribed using Microsoft Word 2019 software and reviewed, analyzed, and extracted concepts several times. The text of each interview was divided into semantic units. Related semantic units were then coded and separated into main categories and sub-categories by examining the differences and similarities between the extracted codes.

This approach combined common keywords in infodemic management and health to develop relevant questionnaire items. Then the items were judged by the research team. After forming the pool of items, the items were removed and reduced and purified. The items that led to distortion of the response pattern were also removed. Following this step, a preliminary version of the questionnaire consisting of 53 items was prepared to undergo psychometric evaluation.

#### Assessing the psychometric properties of the questionnaire

To evaluate the psychometric properties of the questionnaire, assessments of quantitative and qualitative face validity, content validity, construct validity, and reliability were conducted for KAPIM.

##### Face validity assessment

Face validity evaluates whether a tool appears to measure its intended construct [16]. Both qualitative and quantitative methods were employed to evaluate the face validity of KAPIM. The preliminary draft of the questionnaire was subjected to qualitative evaluation by 15 individuals similar to the target group. These participants assessed the difficulty, generality, and ambiguity of the items. The impact scores of the items were then computed to quantitatively assess face validity. During this phase, the participants rated each item on a 5-point Likert scale from completely important to not at all important, with a rating of 5 to 1. Items with an impact score of more than 1.5 were deemed appropriate and retained for subsequent stages [17]. To assess qualitative face validity, 15 key experts were invited to provide feedback on the questionnaire items with regard to their wording, grammar, location in the scale, choice of vocabulary, appropriateness, and scoring [18].

##### Content validity assessment

During the evaluation of content validity, both the Content Validity Ratio (CVR) and the Content Validity Index (CVI) were computed. The CVR was determined by a panel of 10 experts who rated each item’s necessity as “necessary,“ “not necessary but useful,“ or “not necessary.“ As per Lawshe’s Table [19], a CVR value greater than 0.49 for 15 individuals indicates an item’s necessity at a statistically significant level (P = 0.05).The Content Validity Index (CVI) was assessed by the same 15 experts using a 4-point Likert scale to rate questionnaire items based on simplicity, relevance, and clarity, following Waltz & Bausell’s content validity index [20].

##### Construct validity

The Exploratory Factor Analysis (EFA) technique was utilized to evaluate the construct validity of KAPIM and refine the questionnaire’s content for the most concise representation of its underlying components [18].

##### Exploratory factor analysis (EFA)

EFA was carried out through a cross-sectional study. Prichta et al. (2013) stated that the required number of respondents for EFA is 3–10 per item or 100–200 total respondents [18]. Therefore, 250 health care worker were recruited to complete the online questionnaires received from several popular online social networks such as WhatsApp, Instagram, and Telegram or its Iranian equivalent (e.g., Soroush, and Eitta). Data collection was done from January to March 2023., using convenience sampling. No question were excluded from data entry from a total of 250 completed questionnaires. EFA was performed by the principal components method with varimax rotation and using IBM SPSS Statistics V21.0, and the indices used were the Kaiser-Meir-Olkin (KMO) index and Bartlett’s test of sphericity. KMO index indicates sampling adequacy and sufficient sample size to perform factor analysis. The value of this index is between zero and one, and the acceptable value for KMO is more than 0.5. Bartlett’s sphericity test was used to ensure the appropriateness of the data, which measures the significance of the data analysis and was considered at a significance level of 0.95. Three key indicators of eigenvalues, the ratio of explained variance, and scree plot were used to examine the amount and nature of KAPIM tool factors. The scree plot shows the explanatory factors of the factor structure. Based on this diagram, the number of 3 factors in the data was determined. For each component, the item with a factor load of 0.4 and above was kept.

### Reliability assessment

**Fig. 1:**
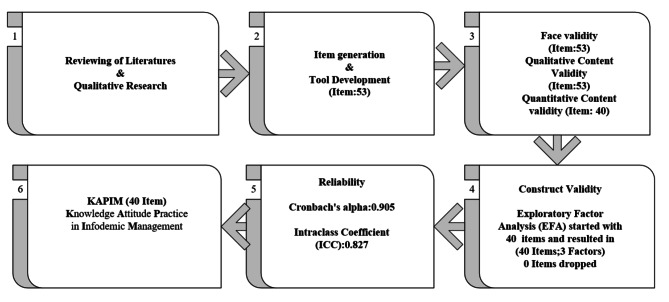
A brief overview of the key stages involved in the psychometric properties of KAPIM

In this study, the Cronbach’s alpha reliability index was computed to assess internal consistency. A satisfactory level for this index was determined to be 0.70 or higher [21]. Figure 1 illustrates a set of instructions outlining the process of developing and evaluating the psychometric characteristics of KAPIM tool.

### Questionnaire description: scaling and scoring

The questionnaire comprises two main components:

1. The initial segment comprises questions that pertain to demographic factors: Gender, Age, Educational level, Educational major, Job, and Work experience (year).
2. The second part of the questionnaire is focused on KAPIM-related inquiries. Its purpose is to evaluate the healthcare workers’ KAP in managing infodemics.

## Result

The results are presented in two distinct sections, namely: (a) **the demographic characteristics of the study participants** and (b) the evaluation of psychometric properties of the KAPIM,

### Demographic results of participants

The data show the demographic and professional characteristics of 250 HCWs. In terms of gender, 82% were female and 18% were male. The majority (41.2%) were aged between 31 and 40 years followed by 20–30 years (30.8%). Most HCWs had a Bachelor’s degree (69.2%), and the most common educational major was Public Health (44.8%), followed by Midwife (36%). In terms of work experience, 31.6% had less than 5 years of experience, while only 0.4% had more than 30 years of experience. The most common job among HCWs was Health Care (50.4%), followed by Midwife (18.4%) and Public Health Expert (14.8%). More details are shown in Table 1.

**Table 1:** Demographic characteristics of the participants in the construct validity

	Variable	Number (percentage)		Variable	Number (percentage)
**Gender**	Man	45 (18)	**Work Experience**	Less than 5 years	79 (31.6)
	Female	205 (82)		6 to 10 years	65 (26)
**Age**	20–30	77 (8.30)		11 to 15 years	36 (14.4)
	31–40	103 (41.2)		16 to 20 years	40 (16)
	41–50	62 (24.8)		21 to 25 years	14 (5.6)
	51–60	7 (2.8)		26 to 30 years	15 (6)
	Above 60 years	1 (0.4)		More than 30 years	1 (0.4)
**Education level**	Diploma	13 (5.2)	**Educational Major**	Health care	20(8)
	Associate Degree	42 (16.8)		Family health	16 (6.4)
	Bachelor’s degree	173 (69.2)		Health Education	77.8)
	MSc	22 (8.8)		Midwife	90 (36)
**Job**	Community Health workers	25 (10)		Nursing	2 (0.8)
	Family health	11 (4.4)		Occupational health	1(0.4)
	Health care	126 (50.4)		Old health	2 (0.8.)
	Health Education	5 (2)		Public health	112(44.8)
	Midwife	46 (18.4)		**Total : 250 HCWs**	
	Public health expert	37 (14.8)			

### Assessment of psychometric properties of KAPIM

In the quantitative component, the report details the assessment of KAPIM face, content, construct validity, and reliability.

#### Item generation

The pool of items in this study comprised a total of 125 items, of which 73 items were generated from the literature review and 53 items was extracted from the codes obtained from interviews with the 17 participants a total duration of 714 min (an average of 42 min per participant) resulted in three main categories (knowledge, attitude, and practice related to infodemic management) and sub-categories (info-rumor sources, perceived myths and rumors, and rumor capturing and info-seeking behaviors).

### Validity

#### Content validity

The content validity ratio (CVR) of five items was less than 0.49, resulting in their removal from the tool. Two items also had a content validity index (CVI) of less than 0.79 and were eliminated. Additionally, six items failed to meet the minimum score requirements for both CVR and CVI and were subsequently removed. The overall CVI of the tool was 0.94. After conducting the content validity assessment, 13 items were removed, leaving the tool with 40 items.

#### Face validity

After evaluating the content validity of the tool and making necessary modifications, both quantitative and qualitative face validity were conducted. After receiving feedback from the participants, necessary modifications were made to 8 items, and since the impact score of all items was higher than 1.5, no item was removed.

#### Construct validity

**Fig. 2:**
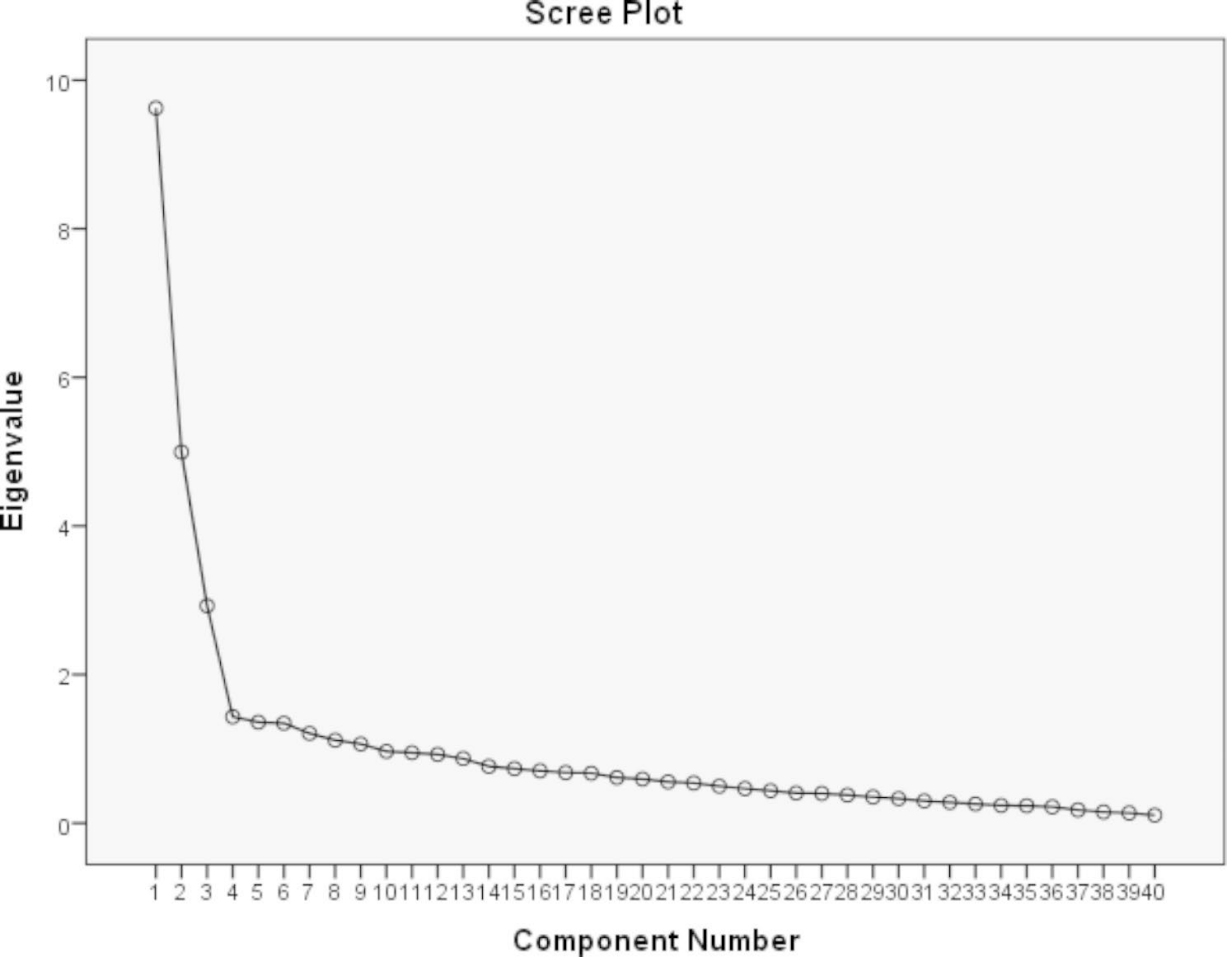
Scree plot of the exploratory factor analysis of KAPIM

To evaluate the construct validity, 250 completed questionnaires were used. Based on Bartlett’s and KMO tests, the results indicate the appropriateness and adequacy of the data for factor analysis, with values of 4996.3 and 0.861, respectively. Based on the results of the exploratory factor analysis(EFA), items with a factor loading of 0.3 or higher were deemed appropriate and no items were removed. At the end of EFA, the tool was left with 40 items. The factor loadings of items related to the extracted factors are presented in Table 2 and Fig. 2. The KAPIM tool for assessing the healthcare workers’ KAP in infodemic management was refined to include 40 items and three factors. Factor 1 comprises 24 items and assesses the knowledge of HCWs related to rumor and infodemic management. Factor 2, consisting of 8 items, focuses on the evaluation of healthcare providers’ attitudes toward infodemic management. Factor 3 includes 8 items and assesses the practice of HCWs in managing rumors and infodemics.

**Table 2:** Summary results of exploratory factor analysis fit statistics

	Items	Factor 1	Factor2	Factor3
**Factor Knowledge**	Rumor is a report of an uncertain or suspicious truth circulating	0.416		
	Knowing the principles of risk communication is part of managing an infodemic.	0.546		
	Uncertainty about information is the basis for creating rumors	0.498		
	An infodemic is the spread of both accurate and inaccurate information on a wide scale.	0.425		
	Managing infodemics is an integral part of managing an epidemic.	0.420		
	Social listening is a step towards identifying accurate sources of information.	0.416		
	Inoculation of health information is part of infodemic management.	0.585		
	Social listening to the concerns and anxieties of a community is the first step in infodemic management.	0.551		
	Infodemic management includes four stages: listening to concerns, enhancing risk perception, resilience to false information, and community interaction and empowerment.	0.568		
	Rumors thrive in situations where people are eager for news and cannot receive it from a reliable source.	0.536		
	Access to credible information sources helps in managing rumors.	0.484		
	A vigilant messaging team helps identify rumors in a timely manner.	0.595		
	Creating a system to detect and monitor messages will help identify rumors early.	0.534		
	Sorting and prioritizing rumors in the message identification phase is necessary for infodemic management.	0.564		
	Social participation in identifying rumors is helpful.	0.581		
	Responding to rumors requires the use of various information sources (experts, community members, mass and social media).	0.632		
	Universal education in credible media is necessary for correcting rumors.	0.666		
	Attention to community values and traditions is essential in responding to rumors.	0.606		
	Public and widespread access to information and educational materials must be provided for rumor management.	0.554		
	Managing anxiety, fear, shock, anger, and disbelief is important in responding to rumors.	0.558		
	Providing honest and transparent information is essential in responding to rumors.	0.592		
	Forming a rapid content production team is necessary for responding to rumors.	0.540		
	The speed of the rumor monitoring team is essential.	0.482		
	Knowing the principles of risk communication helps in managing rumors.	0.758		
**Factor-2 Attitude**	Rumor management is divided into two active and passive categories, in both cases, it should be dealt with knowledge and experience.		0.708	
	Providing accurate information from credible sources helps in managing rumors.		0.862	
	Increasing public trust in accurate sources of information prevents rumors from spreading.		0.844	
	Media literacy in the field of health helps in managing infodemics.		0.785	
	Updating information and evidence helps in managing rumors.		0.836	
	Familiarity with the culture, beliefs, and concerns of people helps in managing rumors.		0.826	
	Using social listening techniques can help in managing rumors in the field of health.		0.752	
	If I receive information about health and disease, I share it with those around me.		0.510	
**Factor-3 Practice**	If I receive information about health, I ask the relevant expert about its accuracy.			0.670
	If I receive information about health, I check its validity in credible scientific sources (websites, reference books, articles, etc.).			0.626
	If I am confident that the information about health is incorrect, I inform the last sender.			0.722
	If I am confident that the information about health is incorrect, I share the correct information.			0.711
	In field monitoring, I listen to the information, beliefs, and concerns of people.			0.684
	I am flexible about incorrect information, beliefs, and concerns of people.			0.626
	I record and take notes of false messages, rumors, and incorrect beliefs in the field of health.			0.819
	I document my learned lessons and social experiences in dealing with incorrect information and rumors.			0.807

### Reliability

In the initial phase, the overall reliability of the tool was reported as adequate with a Cronbach’s alpha of 0.905 The effect of removing each item on Cronbach’s alpha was also assessed and showed that deleting individual items had minimal impact on the overall alpha coefficient. The ICC of the entire tool was calculated as 0.8270 (Table 3)

**Table 3:** Cronbach’s alpha and test-retest results

Domeinn	Items	Cronbach’s alpha (N = 250)	ICC(N = 15)	The total eigenvalue	Percentage of variance	Cumulative variance
Knowledge	24	0.884	0.887	9.62	24.06	24.06
Attitude	8	0.936	0.936	4.99	12.48	36.545
Practice	8	0.875	0.875	2.92	7.31	43.858
**Total**	**40**	**0.905**	**0.827**			

#### Scoring

The scale was designed to assess three areas: knowledge, attitude, and practice. For the knowledge area, participants were presented with three response options (true, don’t know, false) and asked to assign a score between 1 and 3. In the attitude area, participants were given five response options (completely agree, agree, indifferent, disagree, completely disagree) and asked to rate each statement on a score of 1 to 5. Finally, the practice area was evaluated with five response options (always, often, sometimes, rarely, never), and a score from 1 to 5 was assigned to each option.

## Discussion

This study designed and validated a tool that assesses HCWs’ KAP regarding infodemic management. The tool is designed to be comprehensive and covers various aspects of infodemic and rumor management, including identifying and addressing misinformation, understanding effective communication strategies, and responsibly using social media. Public health emergencies can be overwhelming and leave the world vulnerable to misinformation, malinformation, and rumors. As the first line of healthcare providers during public health emergencies, it is crucial that HCWs worldwide possess sufficient knowledge about all aspects of these emergencies. They play a significant role in managing infodemics and can help prevent further damage caused by the spread of inaccurate information. Preparing for disasters and information crises, understanding the risk or risk perception, and having the right knowledge are important factors of infodemic management that should be institutionalized in HCWs [22].

### Knowledge about infodemic management

The KAPIM tool identifies knowledge as the first factor, consisting of 24 items. This factor assesses healthcare workers’ knowledge in managing infodemics, including the fundamentals of infodemic management and the factors that affect its identification and management. Healthcare providers can better assess the effectiveness of their messaging and determine whether they are reaching their intended audience by understanding the dimensions of infodemic management. This includes assessing if communities are being sensitized to health practices and if health messages are appropriately reaching marginalized communities. It is crucial for HCWs to understand the different types of infodemics to effectively manage them [4].

The published findings and protocols of the World Health Organization support this study’s conclusion that healthcare workers must possess adequate knowledge to identify different types of infodemics, including misinformation, misinformation, rumors, and disinformation. This knowledge is necessary to effectively plan, compile, and implement infodemic management strategies. In many countries, maintaining and promoting public trust is crucial. This involves not only providing clear and timely information but also engaging in two-way communication and addressing the public’s understanding of risk and needs. Proactively sharing information and filling gaps in knowledge from before a public health emergency to the rehabilitation phase can increase public trust and aid in managing infodemics, social networks, and rumors. This can help alleviate stress and confusion caused by emergencies and disasters [23].

Within the domain of knowledge, information management is a crucial component [24]. It is emphasized that responding to rumors requires the use of various information sources, including experts, community members, and mass and social media. Additionally, providing public and wide access to information and educational materials is essential in managing rumors. To address rumors, it is imperative to provide truthful and transparent information. To achieve this, a rapid content production team must remain active and up-to-date.

### Attitude about infodemic management

This tool measures the perspectives of HCWs toward key strategies in managing an Infodemic, consisting of eight items. The study highlighted the importance of evaluating and understanding community ecology for the effective management of infodemics and rumors. To assess public opinion and the ecology of health communication, healthcare providers should use social intelligence techniques to help manage health rumors [25]. To develop an effective strategy for managing an Infodemic, it is crucial to evaluate the audience and those impacted in terms of literacy, social, economic, cultural, and societal values and responses. Social participation plays a crucial role in identifying and dispelling rumors as it helps educate people on reliable media sources and the importance of correcting misinformation with the help of healthcare providers [26].Effective planning and timely execution by healthcare workers can enhance public trust in the right sources of information, which can prevent rumors from spreading and promote accurate dissemination of health-related information [27, 28].

### Practice in infodemic management

The third factor of KAPIM measures the practice of HCWs despite infodemics and rumors with 8 items. How to evaluate news and information, search for the primary source, and correct and not repost misinformatins is considered in this factor. Based on Kenneth’s study, there are ways to get information. The coordination that Kenneth Lee et al(2014) state is that health information is ubiquitous on the Internet and frequently used by health consumers. Finding and understanding online health information, and determining the reliability of the content, pose real challenges for many health consumers [29].

Previous research has identified several strategies for obtaining reliable health information. In one study, Kenneth Lee et al. (2014) found that health information is widely available online and frequently used by health consumers. However, accessing and interpreting online health information and determining its trustworthiness can be challenging for many consumers.According to a study conducted by Jude Alawa et al. (2021), providing healthcare workers with adequate information about COVID-19 can improve patient outcomes and reduce the spread of the disease. It is also important for healthcare workers to have appropriate knowledge and attitudes regarding health communication, rumors, and community conditions [13]. Furthermore, Limaye et al. (2020) conducted a study on the management of social networks for infodemic management. Their findings highlight the importance of various groups, such as government leaders, social media companies, and healthcare providers, in responding to the challenges of infodemics. Healthcare providers, in particular, play a crucial role in preventing the use of social media to spread misinformation and distrust, which can endanger public health [14].

This research marks an initial step toward evaluating the knowledge, attitude, and practice of healthcare workers (HCWs) in managing infodemics, an area that, to the best of our knowledge, has not been fully developed. Thus, the study contributes to a growing body of evidence supporting the effectiveness of a valid and reliable tool in assessing infodemic management, with a focus on domains essential to the cognitive process of health behavior. However, the discussion could have been more nuanced and comprehensive if comparable studies were available to compare findings. Therefore, to gain a better understanding of the sensitivity and accuracy of KAPIM, it is crucial for the tool to be tested in multiple interventional studies. In addition, we recommend that the tool be tested on individuals with varying levels of education and professions.

## Conclusion

In conclusion, the study used a rigorous process for item generation, validation, and reduction; the KAPIM tool assesses three critical domains of healthcare workers’ KAP related to infodemic management, which can inform targeted interventions to improve healthcare workers’ preparedness and response to infodemics. Therefore, the KAPIM tool is valuable for assessing and improving the knowledge, attitude and practice of healthcare workers in infodemic management. The development and validation of the KAPIM tool represent a significant step forward in infodemic management and have important implications for healthcare organizations and policymakers seeking to address the challenges posed by the rapidly evolving infodemic landscape. In addition to assessing the cap of health workers, this tool can help policymakers plan according to the current situation, gaps, and educational needs. Training HCWs according to this tool’s components and dimensions can help them manage rumors in public health emergencies.

## Data Availability

The datasets used and/or analysed during the current study available from the corresponding authoron reasonable request.
